# Curcumin Represses NLRP3 Inflammasome Activation via TLR4/MyD88/NF-κB and P2X7R Signaling in PMA-Induced Macrophages

**DOI:** 10.3389/fphar.2016.00369

**Published:** 2016-10-10

**Authors:** Fanqi Kong, Bozhi Ye, Jiatian Cao, Xueli Cai, Lu Lin, Shanjun Huang, Weijian Huang, Zhouqing Huang

**Affiliations:** ^1^The Key Lab of Cardiovascular Disease of Wenzhou, Department of Cardiology, The First Affiliated Hospital of Wenzhou Medical UniversityWenzhou, China; ^2^Division of Cardiology, Shanghai Ninth People’s Hospital Affiliated Shanghai Jiaotong University School of MedicineShanghai, China

**Keywords:** Curcumin, NLRP3 inflammasome, TLR4/MyD88/NF-κB, P2X7R, macrophages

## Abstract

**Aims:** In the NOD-like receptor (NLR) family, the pyrin domain containing 3 (NLRP3) inflammasome is closely related to the progression of atherosclerosis. This study aimed to assess the effects of curcumin on NLRP3 inflammasome in phorbol 12-myristate 13-acetate (PMA)-induced macrophages and explore its underlying mechanism.

**Methods:** Human monocytic THP-1 cells were pretreated with curcumin for 1 h and subsequently induced with PMA for 48 h. Total protein was collected for Western blot analysis. Cytokine interleukin (IL)-1β release and nuclear factor kappa B (NF-κB) p65 translocation were detected by ELISA assay and cellular NF-κB translocation kit, respectively.

**Results:** Curcumin significantly reduced the expression of NLRP3 and cleavage of caspase-1 and IL-1β secretion in PMA-induced macrophages. Moreover, Bay (a NF-κB inhibitor) treatment considerably suppressed the expression of NLRP3 inflammasome in PMA-induced THP-1 cells. Curcumin also markedly inhibited the upregulation of toll-like receptor 4 (TLR4), myeloid differentiation factor 88 (MyD88), phosphorylation level of IκB-α, and activation of NF-κB in PMA-induced macrophages. In addition, purinergic 2X7 receptor (P2X7R) siRNA was administered, and it significantly decreased NLRP3 inflammasome expression in PMA-induced macrophages. Furthermore, curcumin reversed PMA-stimulated P2X7R activation, which further reduced the expression of NLRP3 and cleavage of caspase-1 and IL-1β secretion. Silencing of P2X7R using siRNA also suppressed the activation of NF-κB pathway in PMA-induced macrophages, but P2X7R-silenced cells did not significantly decrease the expression of TLR4 and MyD88.

**Conclusion:** Curcumin inhibited NLRP3 inflammasome through suppressing TLR4/MyD88/NF-κB and P2X7R pathways in PMA-induced macrophages.

## Introduction

Atherosclerosis is a chronic and progressive immunoinfla mmatory disease. Monocytes are one of major factors in the development of this disease. Focal recruitment of circulating monocytes is one of the earliest cellular responses, which underlie disease progression. Moreover, inflammatory factors, which are released by the newly differentiated macrophages, play key roles in the pathophysiology of atherosclerosis (Glass and Witztum, 2001; Libby, 2002; Hansson, 2005).

For the NOD-like receptor (NLR) family, the pyrin domain containing 3 (NLRP3) inflammasome plays a crucial role in the inflammatory response (Schroder and Tschopp, 2010). NLRP3 inflammasome is a multiprotein complex that consists of NLRP3, an apoptosis-associated speck-like protein containing a caspase recruitment domain (ASC) and caspase-1 (Latz et al., 2013). Upon stimulation, NLRP3 recruits its adaptor ASC and pro-caspase-1 to form an inflammasome complex; consequently, caspase-1 is activated, which causes the cleavage of the pro-forms of interleukin (IL)-1β and IL-18 to their mature forms (Martinon et al., 2009). Moreover, cholesterol crystals induce NLRP3 inflammasome activation and IL-1β secretion in human macrophages (Duewell et al., 2010; Rajamaki et al., 2010). IL-1β is a fundamental pro-inflammatory cytokine in mediating atherosclerosis progression (Elhage et al., 1998; Duewell et al., 2010).

Curcumin, a natural polyphenolic compound in *Curcuma longa*, exhibits various biological properties, including anti-inflammatory, antioxidant, and anti-infection (Shishodia, 2013). Briefly, curcumin administration has been previously associated to regulate different inflammatory cytokines, such as IL-1β (Sun et al., 2013), extracellular matrix metalloproteinase inducer, and matrix metalloproteinase-9 expression (Cao et al., 2014). In addition, the effect of curcumin is associated with the inhibition of different signaling pathway activations, including the activation of toll-like receptor 4 (TLR4), nuclear factor kappa B (NF-κB), and mitogen-activated protein kinase pathways (Min et al., 2013; Zhou et al., 2015). However, whether the anti-atherogenic effects of curcumin involve in suppressing NLRP3 inflammasome activation has never been indicated.

Hence, the present study aims to: (i) identify the expression of NLRP3 inflammasomes under curcumin treatment in monocytes/macrophages; and (ii) elucidate the relative mechanism of curcumin treatment on the inflammatory activity of monocytes/macrophages.

## Materials and Methods

### Cell Culture

Human monocytic cell line (THP-1) was obtained from the American Type Culture Collection (Rockville, MD, USA) and maintained at a density of 10^6^/ml in RPMI 1640 medium containing 10% fetal bovine serum (FBS), 10 mM 4-(2-hydroxyethyl)-1-piperazineethanesulfonic acid (HEPES) (Sigma–Aldrich, USA), and 1% pen/strep solution at 37°C in a 5% CO_2_ incubator. The cells were cultured in 6-well plates for 48 h in the presence of 100 nM phorbol 12-myristate 13-acetate (PMA), which allowed them to differentiate into adherent macrophages. Cells were stimulated for 1 h with curcumin (0–50 μM, Sigma–Aldrich, USA) or 5 μM Bay 11-7082 (NF-κB-specific inhibitor; Beyotime Biotech, China) and subsequently treated with PMA for another 48 h.

### Determination of Cell Viability (CCK8 Assay)

CCK8 assay (WST-8, Dojindo, Kumamoto, Japan) was used to evaluate the cytotoxicity of curcumin on PMA-induced macrophages, according to the manufacturer’s recommendation. PMA-induced macrophages were seeded in 96-well plates at 6 × 10^3^ cells/well. At 24 h later, cells were incubated with curcumin (0–100 μM) for 48 h.

### siRNA Transfection

Cells were transfected with 20 nM of siRNA for 8 h with siRNA transfection reagent (RiboBio, Guangzhou, China) to knockdown purinergic 2X7 receptor (P2X7R). Briefly, cells were treated with 100 nM of PMA for 48 h and washed in fresh medium without antibiotics. Afterward, the cells were treated with siRNA duplex solution for 8 h. The medium was subsequently replaced with normal culture medium. Control cells were transfected with scrambled sequence siRNA control (RiboBio, Guangzhou, China). The cell lysates were utilized for Western blot analysis to verify the efficacy of protein knockdown by siRNA.

### Western Blotting

Protein isolation and Western blot analysis were performed comparably, as described in literature (Huang et al., 2011). Briefly, membrane protein samples were subjected to sodium dodecyl sulfate–polyacrylamide gel electrophoresis and blotted onto a polyvinylidene difluoride membrane (Bio-Rad, Hercules, CA, USA). The membranes were blocked with 5% milk at room temperature and incubated at 4°C overnight with NLRP3, IL-1β, P65, phospho-P65, P-IκB-α, IκB-α, GAPDH, P2X7R, Bax, Bcl-2 (Cell Signaling Technology, Boston, MA, USA) (1:1000 dilution in TBST) or TLR4, and MyD88 (Abcam, Cambridge, MA, UK) (1:500 dilution in TBST) or caspase-1 (Santa Cruz, CA, USA) (1:500 dilution in TBST). Proteins were visualized with ECL procedure (Bio-Rad, USA). The results were analyzed using Quantity One (Bio-Rad) software.

### ELISA for Cytokine Measurements

Cytokines were measured by ELISA in 48-h culture supernatants. On this basis, cells were preincubated in the presence or absence of curcumin (6.25–25 μM) for 1 h or 5 μM Bay 11-7082 (NF-κB-specific inhibitor) for 30 min. PMA was added to the cells at a final concentration of 100 nM, and the cells were further incubated for 48 h. Culture supernatants were analyzed to determine IL-β concentrations using sandwich enzyme immunoassay kits (R&D Systems Europe Ltd, Abingdon, UK), according to the manufacturer’s instructions.

### Immunofluorescence Staining of NF-κB p65

Human monocytic THP-1 cells were cultured on 20-mm diameter glass coverslips in 12-well plates. Cells were pretreated with curcumin (6.25–25 μM) for 1 h and subsequently treated with PMA (100 nM) or vehicle control for 48 h. The cells were immunofluorescence-labeled using a cellular NF-κB translocation kit (Beyotime Biotech), according to the manufacturer’s protocol. Briefly, after washing and fixing, the cells were incubated with a blocking solution at 4°C overnight and subsequently with the NF-κB p65 antibody for 2 h. After washing thrice, a rabbit IgG antibody conjugated with Cy3 was added and incubated for 1 h. To stain the nucleus, the cells were incubated with 4′,6-diamidino-2-phenylindole (DAPI) for 5 min. The activation of NF-κB p65 was visualized with an inverted fluorescence microscope (Olympus DP70) at excitation wavelengths of 350 and 540 nm for DAPI and Cy3, respectively. The red and blue images were overlaid to create a two-color image, in which purple fluorescence indicated the areas of colocalization.

### Statistical Analysis

All values were expressed as mean ± SEM. One-way ANOVA and subsequent *post hoc* Tukey’s test were employed to analyze the differences between sets of data. Statistics was analyzed using the SPSS 20.0 software. Values of *P* < 0.05 were considered statistically significant.

## Results

### Effects of Curcumin on Cell Viability and Apoptosis

**FIGURE 1:**
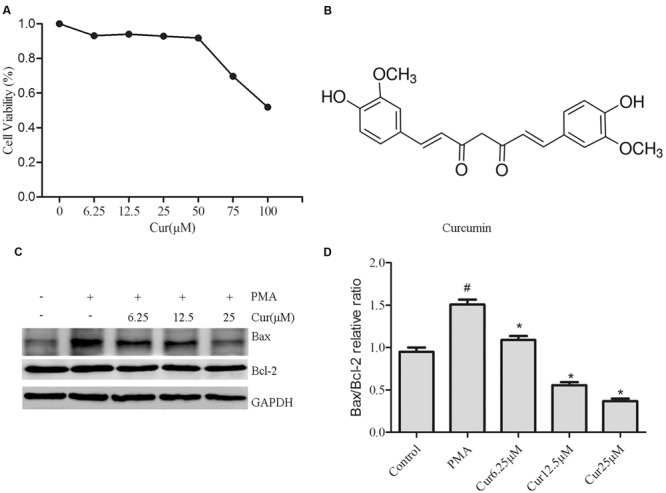
**Effects of curcumin on cell viability and apoptosis.** THP-1 monocytes were incubated with various curcumin concentrations (0–100 μM) for 1 h and exposed to 100 nM of phorbol 12-myristate 13-acetate (PMA) for 48 h. **(A)** Cell proliferation was assessed using the CCK8 assay. Cells incubated in a medium without curcumin and PMA were defined as control and considered to have a 100% proliferation rate. **(B)** Chemical structure of curcumin. **(C)** Representative Western blot analysis of Bax and Bcl-2 in curcumin (6.25–25 μM)-treated THP-1 cells. **(D)** Densitometric analysis was used to quantify the ratio of Bax/Bcl-2. The results represent the mean ± SEM for three experiments. ^∗^*P* < 0.05 vs. PMA group, ^#^*P* < 0.05 vs. Control group.

We first examined the effect of curcumin on the viability of PMA-induced THP-1 cells. PMA-induced macrophages were treated with curcumin (6.25–100 μM) or the vehicle for 48 h. Cell viability was assessed using the CCK8 assay. As shown in **Figure 1A**, curcumin at 50 μM significantly reduced cell viability after 48 h of incubation compared with control ethanol. On this basis, the experiments in cultured THP-1 cells were conducted using 6.25, 12.5, and 25 μM of curcumin. The structure of curcumin used in this study is shown in **Figure 1B**.

To confirm the effect of curcumin on the apoptosis of PMA-induced macrophages, we explored the effect of Bax and Bcl-2 expression by Western blot analysis (**Figures 1C,D**). Significantly, cucurmin-treated cells showed dose-dependent reduction of Bax/Bcl-2 ratio.

### Curcumin Attenuates the Activation of the NLRP3 Inflammasome

**FIGURE 2:**
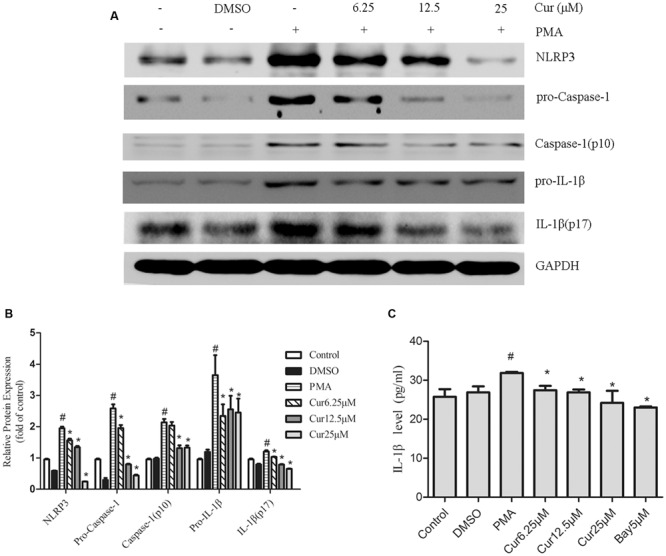
**Curcumin attenuates the activation of the NOD-like receptor (NLR) family, pyrin domain containing 3 (NLRP3) inflammasome.** THP-1 macrophages were stimulated by incubation with curcumin (Cur) at the indicated concentration (6.25–25 μM) for 1 h, followed by PMA for 48 h. The condition referred to as control refers to THP-1 treated with vehicle or dimethyl sulfoxide (DMSO) for 48 h. **(A)** Representative Western blot analysis of NLRP3 and the cleavage of caspase-1 and interleukin (IL)-1β protein expression after PMA-induced inflammasome activation. **(B)** Densitometric analysis was used to quantify the level of NLRP3 and cleavage of caspase-1 and IL-1β. **(C)** Concentrations of IL-1β in cell culture supernatants were detected by ELISA. The results represent the mean ± SEM for three experiments. ^∗^*P* < 0.05 vs. PMA group, ^#^*P* < 0.05 vs. Control group.

To examine the effect of curcumin on NLRP3 inflammasome activation, we stimulated THP-1 cells with PMA in the presence or absence of curcumin. Results displayed that curcumin effectively reduced the cleavage and secretion of IL-1β level in a dose-dependent manner (**Figures 2A–C**). Upon activation, NLRP3, which contains a caspase recruitment domain, causes the cleavage of pro-caspase-1, an essential step to produce and release IL-1β (Schroder and Tschopp, 2010). Consistently, western blot analysis confirmed the reduction of cleaved caspase-1 (p10) and NLRP3 inflammasome level by curcumin (**Figures 2A,B**). These observations suggested that curcumin effectively attenuated the cleavage and secretion of IL-1β level, at least partially, via the inhibition of NLRP3 inflammasome activation.

### NF-κB Pathway is Involved in the Activation of the NLRP3 Inflammasome in PMA-Induced Macrophages

**FIGURE 3:**
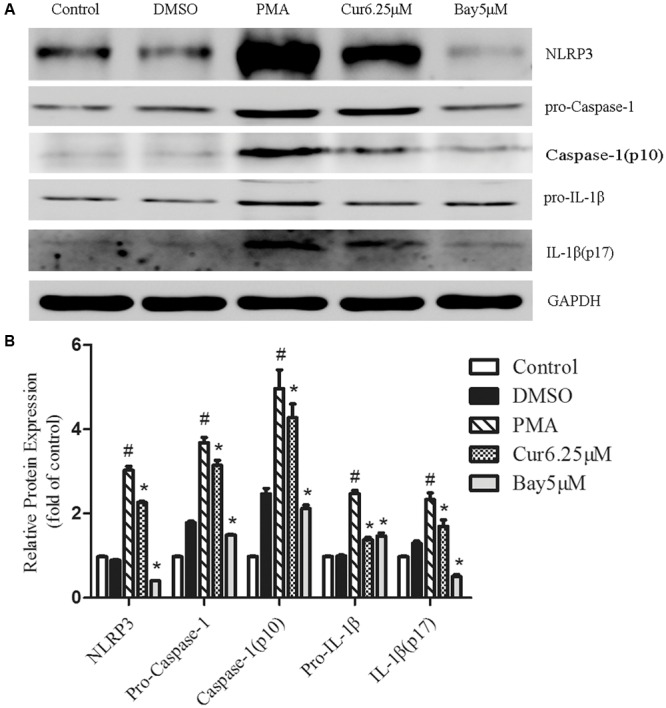
**Nuclear factor kappa B (NF-κB) pathway activation participates in the activation of the NLRP3 inflammasome in PMA-induced macrophages.** Cells were incubated with 6.25 μM of curcumin (Cur) for 1 h or 5 μM of NF-κB specific inhibitor Bay 11-7082 (Bay) for 30 min and exposed to 100 nM of PMA for 48 h before collection. **(A)** Representative Western blot analysis of NLRP3 and the cleavage of caspase-1 and IL-1β protein expression after PMA-induced inflammasome activation. **(B)** Densitometric analysis was used to quantify the level of NLRP3 and cleavage of caspase-1 and IL-1β. The results represent the mean ± SEM for three experiments. ^∗^*P* < 0.05 vs. PMA group, ^#^*P* < 0.05 vs. Control group.

To investigate the associated mechanism of curcumin effect on NLRP3 inflammasome, cells were pre-incubated with 5 μM Bay 11-7082 (NF-κB pathway inhibitor) for 30 min before PMA addition. When cells were cultured in the presence of Bay 11-7082, complete inhibition of NLRP3 expression and cleavage of caspase-1 and IL-1β secretion were observed, which were congruent with the curcumin-pretreated groups (**Figures 3A,B** and **2C**). This result suggested that suppression of NF-κB-signaling pathway activation mitigated NLRP3 inflammasome in PMA-induced macrophages.

### Curcumin Inhibits the Activation of the TLR4/MyD88/NF-κB-Signaling Pathways

**FIGURE 4:**
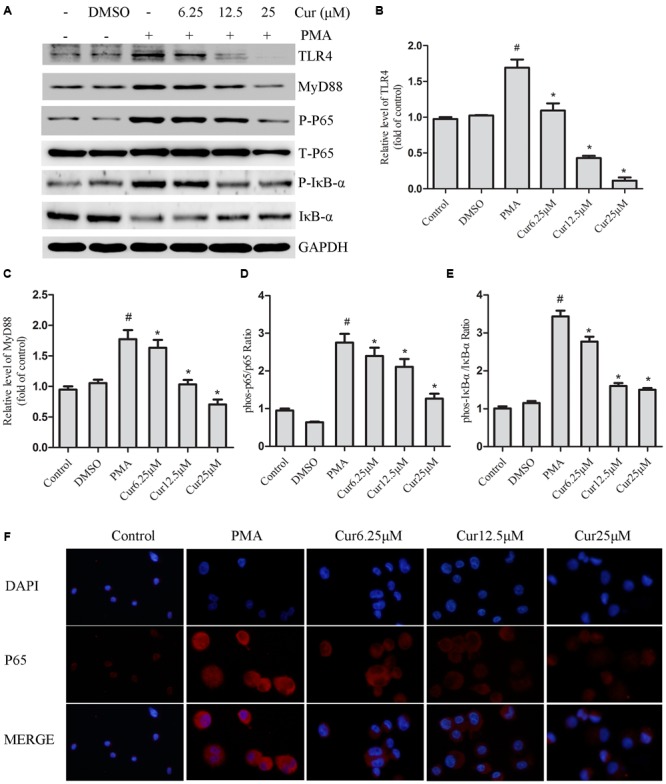
**Curcumin inhibits the activation of the TLR4/MyD88/NF-κB-signaling pathways. (A)** Representative Western blot analysis of TLR4, MyD88, p-IκB-α, IκB-α, p-P65, and P65 was normalized based on the internal control GAPDH. **(B–E)** Densitometry measurements of protein analysis. The results represent the mean ± SEM for three experiments. ^∗^*P* < 0.05 vs. PMA group, ^#^*P* < 0.05 vs. Control group. **(F)** Differentiated THP-1 cells were treated with indicated agent’s immunostained with DAPI (Blue) and anti-NF-κB p65 (Red) and observed using an inverted fluorescence microscope, 200×.

Rapidly growing evidence presented that the TLR4/NF-κB signal transduction pathway is considered an early event essential for inflammasome activation and subsequent IL-1β secretion (Bauernfeind et al., 2009). NLRP3 inflammasome activation during pathological conditions also involves TLR signaling; thus, we monitored the changes in TLR4 expression in PMA-induced THP-1 cells and noticed that PMA stimulation increased the expression of TLR4, which was not observed in control group (**Figure 4A**). Quantification of Western blots showed that TLR4 expression level was reduced in a dose-dependent manner in curcumin treatment group (**Figures 4A,B**). TLR signaling involves the recruitment of MyD88 adapter protein and final activation of NF-κB (Barton and Medzhitov, 2003); hence, we examined the expression of these associated downstream-signaling molecules in cell cultures by Western blot analysis. PMA increased the expression of TLR4 and MyD88, as well as the phosphorylation level of IκB-α and nuclear p65 in the THP-1 cells (**Figure 4A**). Curcumin treatment effectively downregulated the PMA-induced upregulation of TLR4 and MyD88, and phosphorylation level of IκB-α and p65 subunit of NF-κB (**Figures 4A–E**). Additionally, curcumin reduced the nuclear localization of p65 (**Figure 4F**). Taken together, the results implied that curcumin-modulated TLR4/MyD88/NF-κB signalings during PMA stimulation in a dose-depended manner.

### Curcumin Decreases the Expression of P2X7R in PMA-Induced Macrophages

**FIGURE 5:**
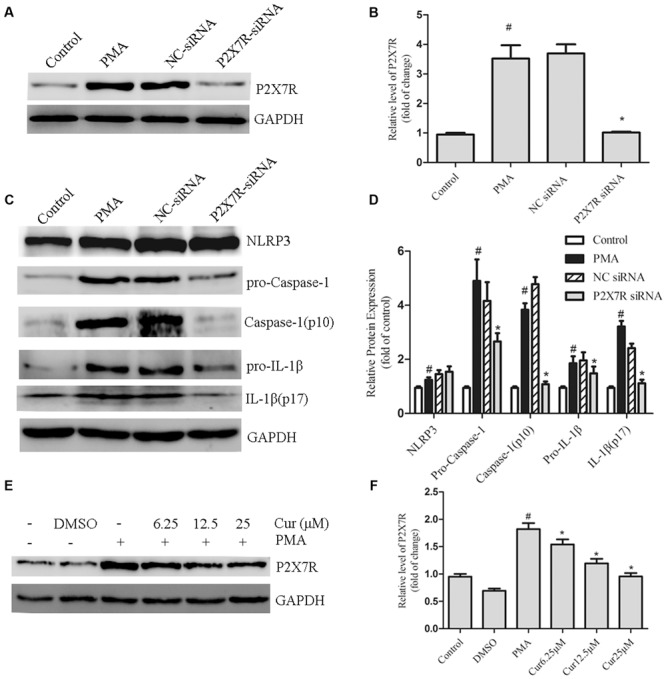
**Curcumin decreases the expression of P2X7R in PMA-induced macrophages. (A,B)** THP-1 cells silenced for purinergic 2X7 receptor (P2X7R) were processed to obtain a whole-cell extract as described under the Section “Materials and Methods”. The expression of P2X7R in cells were transfected with P2X7R or negative control (NC) siRNA. **(C,D)** The expression of NLRP3 and cleavage of caspase-1 and IL-1β in cells were transfected with P2X7R or negative control (NC) siRNA. **(E,F)** THP-1 cells were pretreated with curcumin in the presence of PMA for 48 h. The expression of P2X7R was determined by Western blot analysis. ^∗^*P* < 0.05 vs. PMA group, ^#^*P* < 0.05 vs. Control group.

Purinergic 2X7 receptor is essential for activating the NLRP3 inflammasome (Di Virgilio, 2007). Therefore, we hypothesized that curcumin inhibited the activation of the NLRP3 inflammasome via the mechanism of P2X7R. First, P2X7R siRNA was administered to knock-down the P2X7R expression of PMA-induced macrophages. We successfully and markedly decreased basal P2X7R expression (**Figures 5A,B**). Our data indicated that P2X7R-silenced cells significantly attenuated the activation of caspase-1, which was evidenced by decreased IL-1β production. However, P2X7R-silenced cells did not significantly decrease the expression of NLRP3 (**Figures 5C,D**). Second, we investigated the interaction between curcumin and P2X7R in PMA-induced macrophages. Curcumin inhibited the expression of P2X7R in a dose-dependent manner (**Figures 5E,F**). Our data indicated that curcumin can inhibit the NLRP3 inflammasomes via (at least patially) decreasing P2X7R.

### P2X7R Activation Regulates Nuclear Translocation of NF-κB in PMA-Induced Macrophages

**FIGURE 6:**
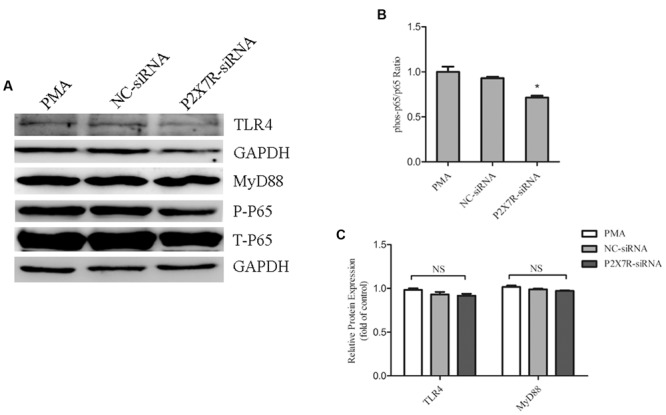
**Purinergic 2X7 receptor inhibition reduces the phosphorylation of p65 in PMA-induced macrophages.** THP-1 cells silenced for P2X7R were used to obtain a whole-cell extract as described under Materials and Methods. **(A)** Representative Western blot analysis of TLR4, MyD88, p-P65, and P65 was normalized based on the internal control GAPDH. **(B,C)** Densitometry measurements of protein analysis. The results represent the mean ± SEM for three experiments. ^∗^*P* < 0.05 vs. PMA group.

To clarify the relationship of P2X7R on the regulation of TLR4/MyD88/NF-κB signalings, we further used P2X7R-silenced cells by siRNA. Our result showed that P2X7R-silenced cells significantly decreased the phosphorylation level of p65 subunit of NF-κB. Nevertheless, the expression of TLR4 and MyD88 (**Figures 6A–C**) was not significantly changed. This finding indicated that P2X7R activation was only involved in regulating the nuclear translocation of NF-κB in PMA-induced macrophages.

## Discussion

In this research, curcumin effectively suppressed the expression of NLRP3 inflammasome in PMA-induced macrophages. Moreover, in PMA-induced macrophages, TLR4/MyD88/NF-κB and P2X7R pathways were necessary for the inhibition of NLRP3 inflammasome expression by curcumin. Therefore, these results supported a novel effect of curcumin on the expression of NLRP3 inflammasome, which suggested curcumin as a promising therapeutic agent for ameliorating the development of atherosclerosis.

Monocytes play a key role in invading atherosclerotic lesions and differentiating them into macrophages (Boyle, 2005; Johnson and Newby, 2009). Plaque-residing macrophages produce chemokines/cytokines, such as IL-1β, which further regulate monocyte/T-cell infiltration into the lesion (Zernecke et al., 2008). PMA-induced IL-1β production is dependent on the NLRP3 inflammasome (Tulk et al., 2015), thereby suggesting that NLRP3 inflammasome can prevent mature IL-1β production. In addition, Zheng et al. (2014) showed that knocking down NLRP3 genes in apolipoprotein E-deficient mice results in reduced inflammatory cytokines and plaque content of lipid and macrophages, as well as increased plaque content of collagen; therefore, NLRP3 is associated with unstable qlaque of atherosclerosis. In our study, PMA treatment-induced NLRP3 inflammasome and the cleavage of caspase-1 and IL-1β expression; moreover, curcumin treatment could markedly mitigate the expression of NLRP3 inflammasome, as well as the cleavage of caspase-1 and IL-1β expression. These findings inferred that curcumin exhibits its anti-inflammatory activity through suppressing the expression of NLRP3 inflammasome in PMA-induced macrophages.

Toll-like receptor 4, which is broadly expressed on the plasma membranes of immune cells, plays a vital role in initiating the sterile inflammation related to atherosclerosis (Guo et al., 2015; Luo et al., 2015). Within the TLR4-signaling pathway, the MyD88-dependent signaling pathway is an important activator of NF-κB and the subsequent regulatory effects of NF-κB signaling (Barton and Medzhitov, 2003). In our study, the expression of TLR4 and MyD88 protein, phosphorylation level of IκB-α and NF-κB p65, and nuclear localization of p65 were significantly increased at 48 h after PMA stimulation compared with the control group. Curcumin treatment obviously suppressed all these indicators. Moreover, Bay 11-7082 (a NF-κB-specific inhibitor) markedly reduced NLRP3 inflammasome activation, which was dramatically attenuated by curcumin treatment. Considering these findings, we suggested the protective effects of curcumin against NLRP3 inflammasome activation by regulating the TLR4/MyD88/NF-κB signaling in PMA-induced macrophages.

Purinergic 2X7 receptor, a member of the P2X subfamily, plays an important role in macrophage regulating cytokine production, which is activated by extracellular ATP to induce NLRP3 inflammasome assembly and caspase-1-dependent processing, and release of pro-inflammatory cytokines IL-1β and IL-18 (Di Virgilio, 2007; Franceschini et al., 2015; Gicquel et al., 2015). P2X7R activation can also activate the NLRP3 inflammasome to promote the progression of atherosclerosis (Piscopiello et al., 2013; Peng et al., 2015). To confirm whether curcumin inhibited NLRP3 inflammasome activation through P2X7R-signaling cascade, the receptor was silenced. As expected, siRNA interference of P2X7R significantly impaired NLRP3 inflammasome activation and IL-1β secretion in PMA-induced THP-1 cells; this result was consistent with the results of Peng et al. (2015). In addition, curcumin significantly inhibited PMA-induced P2X7R expression in macrophages. These results showed that curcumin reduced the activation of NLRP3 inflammasome via regulating P2X7R in PMA-induced THP-1 cells. P2X7R-silenced cells could also decrease the phosphorylation level of p65 subunit of NF-κB, but not TLR4 or MyD88 pathway in PMA-induced THP-1 cells; this observation indicated the activation of p65 is a common downstream of TLR4/MyD88 and P2X7R-signaling pathways. Together, curcumin-downregulated P2X7R expression and TLR4/MyD88/NF-κB signaling regulated the NLRP3 expression, caspase-1 activation, and IL-1β secretion in PMA-induced macrophages.

Indeed previous reports suggest that curcumin can inhibit the NLRP3 inflammasome activation and subsequent release of mature IL-1β both in J774A.1 cells, as well as murine peritoneal macrophages in *in vivo* experiments (Gong et al., 2015). One important difference between our study and that of Gong et al. (2015) is the focus on treatment of cell models. Gong et al. (2015) measured NLRP3 inflammasome production, which was subsequently stimulated with lipopolysaccharide combined with multiple NLRP3 inflammasome activators in mouse macrophages. Additionally, we detected NLRP3 inflammasome production in THP-1 cells stimulated with PMA, which imitated the process of monocyte differentiation to macrophages, which is important for atherosclerosis development. Another important difference is the signaling pathway; they showed that inhibition of NLRP3 inflammasome activation by curcumin involves the downregulation of ERK signaling. We observed that inhibition of NLRP3 inflammasome activation by curcumin involves the downregulation of TLR4/MyD88/NF-κB- and P2X7R-signaling pathways. Altogether, the results of Gong et al. and the present study strongly implicated curcumin as a potent antagonist of NLRP3 inflammasome activation in different pathophysiological processes.

**FIGURE 7:**
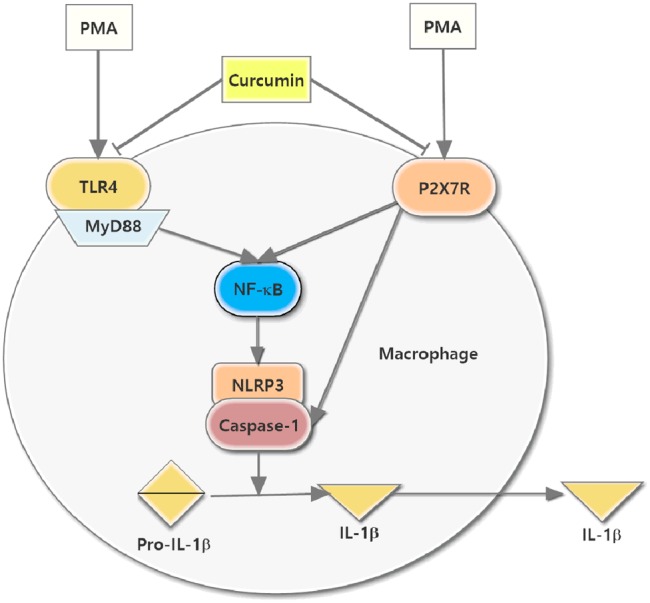
**Schematic model for the reduction of NLRP3 inflammasome expression in PMA-stimulated macrophages treated with curcumin.** The curcumin-downregulated P2X7R expression and TLR4/MyD88/NF-κB signaling, which together inhibited NLRP3 expression, caspase-1 activation, and the secretion of IL-1β.

Given the effect of curcumin on the expression of NLRP3 inflammasome and regulation of TLR4/MyD88/NF-κB and P2X7R, we concluded that curcumin reduced NLRP3 inflammasome, cleaved caspase-1 induction, and consequently reduced IL-1β secretion through TLR4/MyD88/NF-κB and P2X7R pathways in PMA-induced macrophages; the schematic model is shown in **Figure 7**. This study suggested that curcumin might act as an effective candidate for inflammation in atherosclerosis and have advantages for potential clinical applications.

## Author Contributions

FK designed and performed the experiments, analyzed the data, and drafted the manuscript. BY and JC assisted in the experiments. XC, LL, and SH assisted in data analysis. WH revised the paper. ZH designed the study, drafted and revised the manuscript. All authors read and approved the final manuscript.

## Conflict of Interest Statement

The authors declare that the research was conducted in the absence of any commercial or financial relationships that could be construed as a potential conflict of interest.
